# The Leaping Ague of Angus

**Published:** 1829-07-01

**Authors:** 


### LEAPING AGUE OF ANGUS-SHIRE.

Mr. Crichton has recorded a curious case of this kind in the last number of the Edinburgh Journal, which is deserving of notice.

In January, 1818, the author was called to a brisk and lively young girl of 15, of whose history the following are the particulars. In the Summer of 1815, she had suffered from stomach complaints, and great sickness. In October, 1816, thieves broke into the house where she was asleep. She sprang out of bed—leaped out of a window, and roused the people of an adjoining house. This shock was followed by another, the death of a favourite sister.

"She became pensive and bewildered, was affected with excessive perspirations, and her strength rapidly declined. At one period during the Summer the catamenia made a slight appearance but never returned. Towards the close of the year 1817, she had frequent attacks of shaking, accompanied with foaming at the mouth, and followed by a state of coma, which, after continuing about an hour, gradually went off. At the commencement of the year 1818, when my attention was directed to her case, the distress had assumed the following appearances.

Every morning about ten o'clock she became drowsy and torpid; about eleven she began to arouse out of that state; by twelve she got out of bed, and went through the house collecting her trinkets, such as watches, rings, writing apparatus, and other articles she had secreted the preceding day in holes and other bye-places out of sight. These she brought with her into bed, and amused herself with them for some time, occasionally conversing with those in the room, but in such a language that no stranger, and hardly even those of the family, who were constantly beside her, could understand. This arose from her commencing the sentences with the last word, and very frequently pronouncing the words themselves with the last letter foremost. At times, when by no possibility she could make herself be understood by her parents or sisters, she became irritated, and would write down what she wished to convey; but her manner of writing was equally singular, beginning at the right edge of the paper and writing backwards towards the left, the last word of the sentences first, and often the last letter of the word first, and this she performed with great rapidity, and seemingly without consideration. Her sight likewise was peculiarly affected, seeing objects only in particular directions, so that when she wished to view any thing she was necessitated to turn her head in another direction. About one o'clock she again got out of bed, and, after carefully secreting her trinkets in various bye-places of the house, she commenced dancing the *Copenhagen jig.* Her excitations continuing to increase, she jumped upon the tables and chairs, sometimes running with great rapidity round and round the edge of a table, then springing up and squatting herself upon the top of the room door, swinging backwards and forwards without any hold. At this time she required to be very narrowly watched for fear of her springing out of the window, which she often manifested an earnest longing to do. Upon one occasion the outer door happening to be open, she made a sudden spring out, clearing the staircase at one bound. She was instantly followed and brought back without having sustained any injury. At first they were in the habit of attempting to keep her forcibly down in bed, fearing she might injure himself. But the strength of several people together was insufficient for that purpose, as she got out of their hold like an eel, springing to the other end of the room, so that it was thought most advisable to allow her to take her own way, only guarding the windows and door. About two o'clock, becoming quite exhausted, she got into bed, and, falling into a deep sleep, she awakened about five o'clock in her right mind, and without being in the smallest degree conscious of any thing that had taken place during the paroxysm. She continued so until about ten o'clock next morning, when the same or nearly the same routine took place."

Various medicines were used, with little effect; but the disease gradually subsided, and a voyage to the Baltic completely restored the young lady to health.

The term ague is, we conceive, improper. The complaint had nothing of ague about it, except the periodicity, which is not exclusively peculiar to intermittent. The disease was one of those indescribable forms of hysteria, which defy all systems of nosology, all doctrines of pathology—and too often all kinds of remedy, except time. We have, at this moment, under our care, two young ladies whose complaints are, we have no doubt, of this class—but such are the strange forms assumed by the pathological PROTEUS in these two cases, that were we to describe them, we would not be believed. At an earlier period of our experience we should have set them down as deceptions; but we are convinced that these females are driven to their antics by an irresistible propensity—and cannot control their motions or actions. In one case, and that the most distressing and of longest standing, a remarkable amelioration has been produced by remedial agency; but the case we dare not detail, in consequence of the state of a portion of the medical press, which, by misrepresentation and circulation among non-professional readers, will go far to check all confidential communications to the profession, lest injury should be sustained both by patient and practitioner! This is one of the many evils which have flowed, and will yet flow from the system of banishing all honorable principles from medical society. The mischief is coming home to the breast of every reflecting person in the profession.

